# Identifying Contributors to Unmet Community Mobility Needs in Older Adults After Home Physical Rehabilitation

**DOI:** 10.1093/geroni/igaf122.4372

**Published:** 2025-12-31

**Authors:** Lindsey Mathis, Morgan Fique, Jason Falvey, Jasmine Cooper

**Affiliations:** University of Maryland School of Medicine, Baltimore, Maryland, United States; University of Maryland School of Medicine, Baltimore, Maryland, United States; University of Maryland School of Medicine, Baltimore, Maryland, United States; University of Maryland School of Pharmacy, Baltimore, Maryland, United States

## Abstract

Community mobility impairments after major injuries can persist even after completing home physical rehabilitation programs. Neighborhood-level barriers are potential contributors, but their specific impacts on long-term outcomes are unclear. Identifying these relationships can inform care to address mobility deficits in relation to complex neighborhoods. As such, this study explores therapy experiences and neighborhood conditions to identify contributors to community mobility deficits after rehabilitation. Semi-structured interviews were conducted between July 2024 and March 2025 via Zoom or telephone. Interviews were recorded and transcribed. De-identified transcripts were analyzed using inductive thematic analysis. Data were collected until thematic saturation was reached and no new findings arose. Reliability was tested between two independent coders using Cohen’s Kappa (0.74). Five major themes emerged: 1) Restrictive Impacts of Unmet Needs on Community Mobility; 2) Benefits of Targeting Everyday Challenges along with Primary Rehab Diagnoses; 3) Social Networks Enhancing Recovery; 4) Fears of Violence influencing Voluntary Outdoor Mobility, Mitigating Influences of Social Cohesion; 5) Impacts of Comorbidities on Community Mobility. Ultimately, these findings suggest that integrating higher-level social and environmental support systems could further improve outcomes for rehabilitation recipients. Home therapy providers might consider integrating environmental needs into rehabilitation programs—such as facilitating preferred outdoor mobility routes—both during and after home care to better support sustained mobility beyond the home.

